# Supplementary material for: From Inflammation to Oncogenesis: Tracing Serum DCLK1 and miRNA Signatures in Chronic Liver Diseases
Source: Int J Mol Sci. 2024 Jun 12;25(12):6481. doi: 10.3390/ijms25126481 (PMC11203803; doi:10.3390/ijms25126481)
Supplement: Supplementary file 1 [file ijms-25-06481-s001.zip › ijms-3004956-supplementary.pdf]

Supplemental Table 1. Patient Samples Classified by DCLK1 Expression Level

ID	Disease	Group	DCLK1 level	DCLK1 ng/mL
30538-001	Control	A	Low	3.22
30538-002	Control	A	Low	5.46
30538-003	Control	A	Low	5.44
30538-004	Control	B	High	40.64
30538-005	Control	B	High	46.38
30538-006	Fibrosis	C	High	58.28
30538-007	Fibrosis	C	High	54.71
30538-008	Fibrosis	D	Low	13.75
30538-009	Fibrosis	D	Low	13.47
30538-010	Fibrosis	D	Low	10
30538-011	Cirrhosis	E	High	52.15
30538-012	Cirrhosis	E	High	52.15
30538-013	Cirrhosis	E	High	52.15
30538-014	Cirrhosis	F	Low	6.16
30538-015	Cirrhosis	F	Low	2.3
30538-016	Hepatocellular	G	High	55.66
30538-017	Hepatocellular	G	High	53.97
30538-018	Hepatocellular	G	High	46.1
30538-019	Hepatocellular	H	Low	10.96
30538-020	Hepatocellular	H	Low	20.04
